# Family reunion activity may be used as an alternative item for sexual activity in the Duke Activity Status Index

**DOI:** 10.1186/s40981-023-00680-2

**Published:** 2023-12-07

**Authors:** Satoki Inoue

**Affiliations:** Division of Intensive Care, Fukushima Medical University School Hospital, 1 Hikarigaoka, Fukushima City, 960-1295 Japan

## To the Editor

The Duke Activity Status Index (DASI) questionnaire is a tool used to evaluate the functional status of patients undergoing non-cardiac surgery [1], as included in the Japanese guideline on perioperative cardiovascular assessment and management for non-cardiac surgery [2]. It comprises five domains that cover different types of common activities, reflecting a wide range of cardiovascular stresses [1]. These domains include personal care, ambulation, household tasks, sexual function, and recreation (sports activity) [1]. Each domain has between one and four items related to it. Participants answer these items, and positive responses are assigned weights. The DASI score can be converted into metabolic equivalents (METs). The Japanese guideline suggests that if a patient’s self-reported functional capacity is equal to or greater than 7 METs, further preoperative cardiovascular examinations may not be necessary [2].

Assessing sexual function is included as part of evaluating each patient in DASI. However, in Japan, especially among the elderly, cultural norms often lead to patients feeling uncomfortable when asked such private questions. This discomfort around discussing sexual matters is not unique to Japan; globally, the topic of sexual activity is considered sensitive in medical questionnaires [3]. Excluding this domain results in a mere reduction of 5.25 from the DASI score. This modest reduction translates to approximately 0.645 METs, considering the fixed constant of 2.74 (9.6/3.5) in the DASI formula [1]. Although it is also suggested that removing this potentially sensitive question has minimal impact on METs calculation [3], the absence of this evaluation might lead to instances where the functional capacity, in terms of METs, falls below 7, indicating uncertainty regarding the patient’s suitability for non-cardiac surgery [2].

To address this concern, finding an alternative domain for sexual function in the DASI would be beneficial. The 2011 Compendium of Physical Activities suggests that sexual activity ranges from 1.3 to 2.8 METs [4]. An ideal replacement should mirror this MET range and should be independent or minimally overlapping with other DASI domains. Considering activities involving emotions, passion, and family connections, retreats or family reunion activities—such as sitting, relaxing, talking, eating, and playing games with children—could serve as an alternative. These activities align with a MET range of 1.8 to 3.0 [4] and are reflective of emotional aspects similar to family love.

While family love is not synonymous with sexual desire, these activities are more likely influenced by emotions compared to other activities listed in the Compendium of Physical Activities. To validate this substitution, assessing the modified DASI through peak oxygen uptake measurements is necessary. Besides, recognizing the growing concern over social isolation in Japan, there might be instances where this item cannot be evaluated. Nevertheless, I believe this modification provides an accessible and suitable alternative for evaluating functional capacity in patients undergoing non-cardiac surgery.

## Data Availability

Not applicable.

## Abbreviations

DASI: Duke Activity Status Index
MET: Metabolic equivalent
